# Multiple Locus Variable number of tandem repeat Analysis: A molecular genotyping tool for *Paenibacillus larvae*

**DOI:** 10.1111/1751-7915.12375

**Published:** 2016-07-01

**Authors:** Tine Descamps, Lina De Smet, Pieter Stragier, Paul De Vos, Dirk C. de Graaf

**Affiliations:** ^1^ Laboratory of Molecular Entomology and Bee PathologyFaculty of SciencesGhent UniversityGhentBelgium; ^2^ Laboratory of MicrobiologyFaculty of SciencesGhent UniversityGhentBelgium

## Abstract

American Foulbrood, caused by *Paenibacillus larvae*, is the most severe bacterial disease of honey bees (*Apis mellifera*). To perform genotyping of *P. larvae* in an epidemiological context, there is a need of a fast and cheap method with a high resolution. Here, we propose Multiple Locus Variable number of tandem repeat Analysis (MLVA). MLVA has been used for typing a collection of 209 *P. larvae* strains from which 23 different MLVA types could be identified. Moreover, the developed methodology not only permits the identification of the four Enterobacterial Repetitive Intergenic Consensus (ERIC) genotypes, but allows also a discriminatory subdivision of the most dominant ERIC type I and ERIC type II genotypes. A biogeographical study has been conducted showing a significant correlation between MLVA genotype and the geographical region where it was isolated.

## Introduction

The gram‐positive, rod‐shaped, endospore‐forming bacterium *Paenibacillus larvae* is the ethiological agent of American foulbrood (AFB) (Genersch *et al*., 2006), a deadly brood disease of the honey bee (*Apis mellifera*). Very young larvae, below 36 h of age, are most susceptible. Transmission occurs by the oral uptake of the highly resistant spores that are spread inside the colony or between colonies by adult bees that carry them or by interventions of the beekeeper (Lindstrom, 2008; Lindstrom *et al*., 2008). AFB is classified as a notifiable disease in most countries and depending on the local control strategy, sick colonies are destroyed by burning, decontaminated by the shaking method or treated with antibiotics. Hence, *P. larvae* is responsible for considerable economic losses in the beekeeping sector worldwide.

Historically, it was thought that AFB and ‘powdery scale disease’ were caused by different species, *Bacillus larvae* and *Bacillus pulvifaciens* respectively (Katznelson, 1950). After different rounds of reclassification (Ash *et al*., 1991,1993), they were classified as the same species, but split at subspecies level (Heyndrickx *et al*., 1996). In 2006 was demonstrated that both should be classified as the species *P. larvae* without separation at the subspecies level (Genersch *et al*., 2006).

Over the years, different techniques have been developed for genotyping of *P. larvae*, as there are Restriction Endonuclease Fragment Patterns (Djordjevic *et al*., 1994; Alippi *et al*., 2002), rep‐PCR (Alippi and Aguilar, 1998; Genersch and Otten, 2003), Pulsed‐Field Gel Electrophoresis (Wu *et al*., 2005; Genersch *et al*., 2006), Amplified Fragment Length Polymorphism (de Graaf *et al*., 2006), Denaturing Gradient Gel Electrophoresis (Antunez *et al*., 2007) and most recently Multi Locus Sequence Typing (MLST) (Morrissey *et al*., 2015). Of these techniques, only rep‐PCR has commonly been used for genotyping *P. larvae*. Enterobacterial Repetitive Intergenic Consensus (ERIC) rep‐PCR of the bacteria revealed four genotypes (Genersch and Otten, 2003; Genersch *et al*., 2006). Both genotypes ERIC I and ERIC II have a worldwide distribution (Schafer *et al*., 2014; Morrissey *et al*., 2015). Genotypes ERIC III and IV have not only been identified in field for decades, but exist as few isolates in culture collections (Genersch, 2010). ERIC genotyping has the advantage that it splits the species into biological relevant groups which differ in colony morphology and virulence at individual and colony level (Neuendorf *et al*., 2004; Genersch *et al*., 2005, 2006). The sequencing of the genomes of *P. larvae* ERIC I and ERIC II strains (Qin *et al*., 2006; Chan *et al*., 2011; Djukic *et al*., 2014) provided an important step towards the development of better molecular typing methods. The recently developed MLST method (Morrissey *et al*., 2015) ratifies and extends the ERIC typing scheme. Although this MLST is a very useful method for epidemiology and source tracking, its use in diagnostic labs is hampered by its expensive and labour‐intensive sequencing step.

We developed an alternative, equivalent technique called Multi Locus Variable number of tandem repeat Analysis (MLVA) (van Belkum, 1999) for genotyping *P. larvae*. MLVA is a proven highly discriminatory subtyping method used for many pathogens, such as *Mycobacterium tuberculosis* (Mazars *et al*., 2001), *Yersinia pestis* (Klevytska *et al*., 2001), *Staphylococcus aureus* (Malachowa *et al*., 2005), *Erwinia amylovora* (Buhlmann *et al*., 2014) and *Campylobacter jejuni* (Techaruvichit *et al*., 2015). The method has also been used to link genotype information with geographical information to study how bacteria (*Y. pestis*,* Burkholderia pseudomallei*,* Xanthomonas citri pv. citri*,* Bacillus anthracis* and *E. amylovora*) behave within smaller geographical areas or single outbreaks (Girard *et al*., 2004; U'Ren *et al*., 2007; Bui *et al*., 2009; Stratilo and Bader, 2012; Buhlmann *et al*., 2014). The typing method uses the Variation in Number of Tandem Repeats (VNTR) on different loci among the genome to classify the strains (van Belkum, 1999). These variations are caused by slipped strand mispairing (Torres‐Cruz and van der Woude, 2003). VNTRs have been described as fast molecular clocks (van Belkum, 1999). Indeed, since dynamics of VNTRs depend on repeat copy number (Vogler *et al*., 2006), different VNTRs show different clock speeds (Lindstedt, 2005; van Belkum, 2007). This makes the method suitable for phylogenetic and evolutionary studies. In MLVA, different VNTR loci are combined, allowing inspection of phylogenetic relationships among different bacterial strains. Typically, 5 to 6 loci are combined in one multiplex PCR, which can be analysed using electrophoresis. In this paper, we describe the development of a MLVA‐based genotyping protocol for *P. larvae*, and the subsequent typing of a collection of 209 *P. larvae* strains, demonstrating that MLVA has great potential for genotyping *P. larvae* in an epidemiological context.

## Results and discussion

### Identification of tandem repeat regions

We screened three publically available *P. larvae* genomes [BRL 230010 (Qin *et al*., 2006) and its updated sequence B3650 (Chan *et al*., 2011), DSM 25430 and DSM 25719 (Djukic *et al*., 2014)] for tandem repeats using an online software package (*Tandem Repeats Database* at https://tandem.bu.edu/cgi-bin/trdb/trdb.exe; Gelfand *et al*., 2006). BRL 230010 and DSM 25719 belong to the ERIC I genotype, whereas DSM 25430 is an ERIC type II. To permit VNTR analysis on an agarose gel, the search criteria for tandem repeats were set at a size between 15 and 120 bp with 80% sequence match. This resulted in the finding of 40 different tandem repeat loci (Table S1).

The search for suitable VNTRs was continued by a two‐step procedure. Candidate loci were selected based on different criteria *in silico*, and their respective primers were designed. Three criteria were used: (i) the locus had a different copy number in at least two genomes or (ii) it had a pattern size between 15 and 30 or (iii) it had a copy number of more than five units. In a second step the differentiating power of these loci was tested on 13 different isolates. These isolates included the four different ERIC genoytpe and were isolated at different locations. ERIC types III and IV were represented once and ERIC II twice. The first selection criterium resulted in eight candidate loci, of which four were differentiating enough to be retained for implementation in the multiplex PCR, i.e. VNTR A, VNTR C, VNTR F and VNTR G (Table 1). An optimal MLVA procedure includes 5–6 VNTRs and therefore the search for more VTNRs was needed. The second selection criterium rendered six additional loci. Of these six loci, two were retained after testing in the 13 strains: VNTR B and VNTR D (Table 1). Finally, loci with more than five repeat units were screened. This gave two loci to be tested, one of which was retained: VNTR E (Table 1). Thus, in total seven loci were found with enough differentiating power for implementation in the multiplex PCR.

**Table 1:** VNTR primer sequences

VNTR	Length TR	Forward primer	Length 5′	Reverse primer	Length 3′
VNTR A*	19	GAGGGATATACCCCACCTCTTT	5	GGGGAAGTATGATCCCGAAG	17
VNTR B*	21	CCGGAATAATCCGCTTATGA	22	ATCACCAGAGTTGGCGATTC	3
VNTR C*	24	TGGTTTAGGAACCGGTGTTG	47	CACATTAAAGCCTGTGCAGGTA	38
VNTR D*	24	ATCATGGCGGTTGGGATG	2	CACAGGCTCGACAACCACTA	13
VNTR E*	68	TGTTCAATTTTGATTGTTTTGTTCA	73	TATATGGCGGTCGGCTTAAT	2
VNTR F	48	TACCCCAATCTGCCTTGTTG	70	CATGCTCCTGCGTGGTATAA	41
VNTR G	18	GTCATTACGGCCCAGGTG	20	TGAGGCTGCAAAGACAGATG	22

Five VNTR loci (depicted with *) were used to combine in the multiplex PCR. Of each VNTR locus the length, forward primer and reverse primer are given. The distance (in base pairs) between the primer annealing site and the tandem repeat was mentioned as length 5′ and length 3′.

### Multiplex PCR

**Figure 1:**
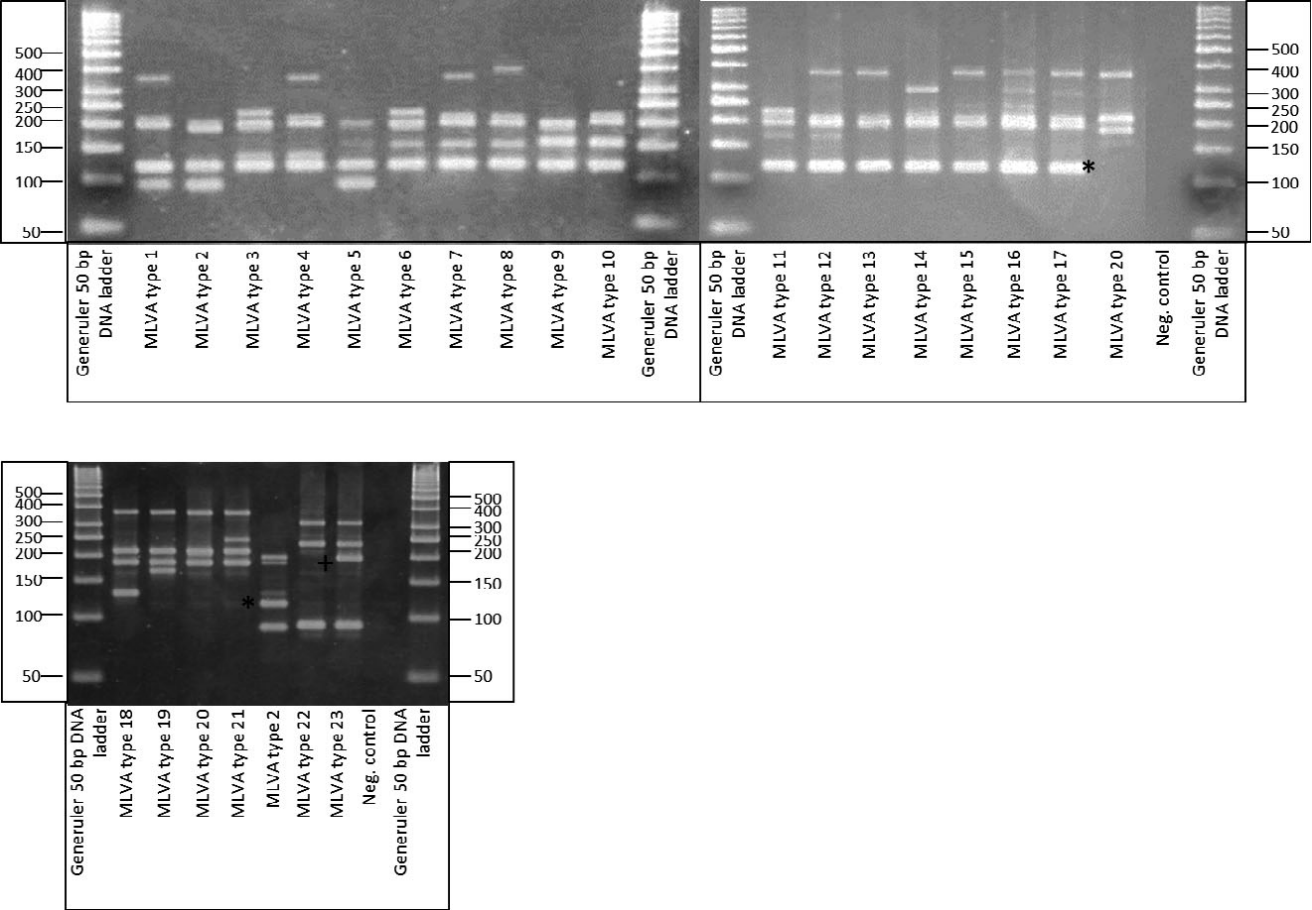
MLVA types. The tested collection of *Paenibacillus larvae* strains contained 23 different MLVA types. The ERIC I types (MLVA type 1–17) all have a band of 120 bp (*), which is absent in all other ERIC types. ERIC III (MLVA type 22) could be differentiated from ERIC type IV (MLVA type 23) by the presence of a 190 bp band (+). ERIC types II and IV do not show an VNTR B amplicon, ERIC type III does not show an VNTR E amplicon.

Optimization of the multiplex PCR was realized by establishing the optimal concentration of MgCl_2_, primers and template DNA. In the final PCR, two loci were omitted because they created non‐target amplicons (VNTR F) or no amplicons during multiplexing (VNTR G). The obtained MLVA profiles were analysed using a 3% High Resolution Agarose Gel (Sigma‐Aldrich, St. Louis, MO, USA) (Fig. 1). The screening of the 13 strains created a total of nine different MLVA patterns. The screening was repeated three times and gave reproducable results.

### MLVA genotyping

The Laboratory of Molecular Entomology and Bee Pathology have access to a large collection of *P. larvae* strains available from the bacteria collection of the Belgian Co‐ordinated Collections of Micro‐organisms (BCCM/LMG) and from its own working collection. The strains that were retained for this study originated from a nationwide honey screening in Belgium (de Graaf *et al*., 2001) (116 isolates) or from clinical outbreaks of AFB in Belgian apiaries between 2013 and 2015 (74 isolates), and were extended with three additional reference strains (BRL 230010; Qin *et al*., 2006; LMG 16252, LMG 16247; both obtained from BCCM/LMG), six Austrian ERIC II isolates (Loncaric *et al*., 2009) and 10 Italian ERIC II strains (Bassi *et al*., 2015). Using these 209 different isolates, 14 new MLVA types were discovered resulting in 23 MLVA patterns.

A representative isolate for each pattern was chosen and its amplicons were sequenced. The resulting sequences confirmed that the primers targeted their respective tandem repeat in each MLVA pattern and that no off‐targets were amplified. The copy size of the tandem repeats could be established for each locus. These were consistent with the size of the amplicon on the gel (after substracting the flanking sequences) (Table 1 and Fig. 1). In Table 2, the typical VNTR‐code is given for the different MLVA patterns. To facilitate the analysis, we attributed a MLVA type number (MLVA 1 – 23) to each unique VNTR combination (Table 2).

**Table 2:** MLVA types

MLVA	VNTR‐code	ERIC genotype	Prevalence (%)
1	2‐6‐4‐3‐5	I	1.0
2	4‐6‐3‐3‐1	I	30.1
3	4‐6‐3‐3‐3	I	1.0
4	4‐6‐4‐3‐5	I	1.4
5	5‐6‐3‐3‐1	I	1.0
6	5‐6‐3‐3‐3	I	3.8
7	5‐6‐4‐3‐5	I	4.3
8	5‐6‐4‐3‐6	I	1.0
9	6‐6‐3‐3‐2	I	2.3
10	6‐6‐4‐3‐2	I	11.0
11	6‐6‐4‐3‐3	I	1.4
12	6‐6‐4‐3‐5	I	1.9
13	7‐6‐4‐3‐5	I	17.7
14	10‐6‐4‐3‐4	I	3.8
15	10‐6‐4‐3‐5	I	4.8
16	13‐6‐4‐3‐4	I	0.5
17	13‐6‐4‐3‐5	I	3.3
18	4‐0‐3‐7‐5	II	0.5
19	6‐0‐3‐7‐5	II	1.0
20	7‐0‐3‐7‐5	II	6.2
21	8‐0‐3‐7‐5	II	0.5
22	2‐6‐5‐11‐0	III	0.5
23	2‐0‐5‐11‐0	IV	0.5

Of each MLVA type the VNTR‐code, ERIC genotype and prevalence in our dataset is given. Seventeen MLVA types belonged to ERIC type I strains, four belong to ERIC type II. ERIC type III and IV were represented by only one MLVA type. Most MLVA types show a very low prevalence, only three types have a prevalence higher than 10%.

### VNTR characteristics

**Figure 2:**
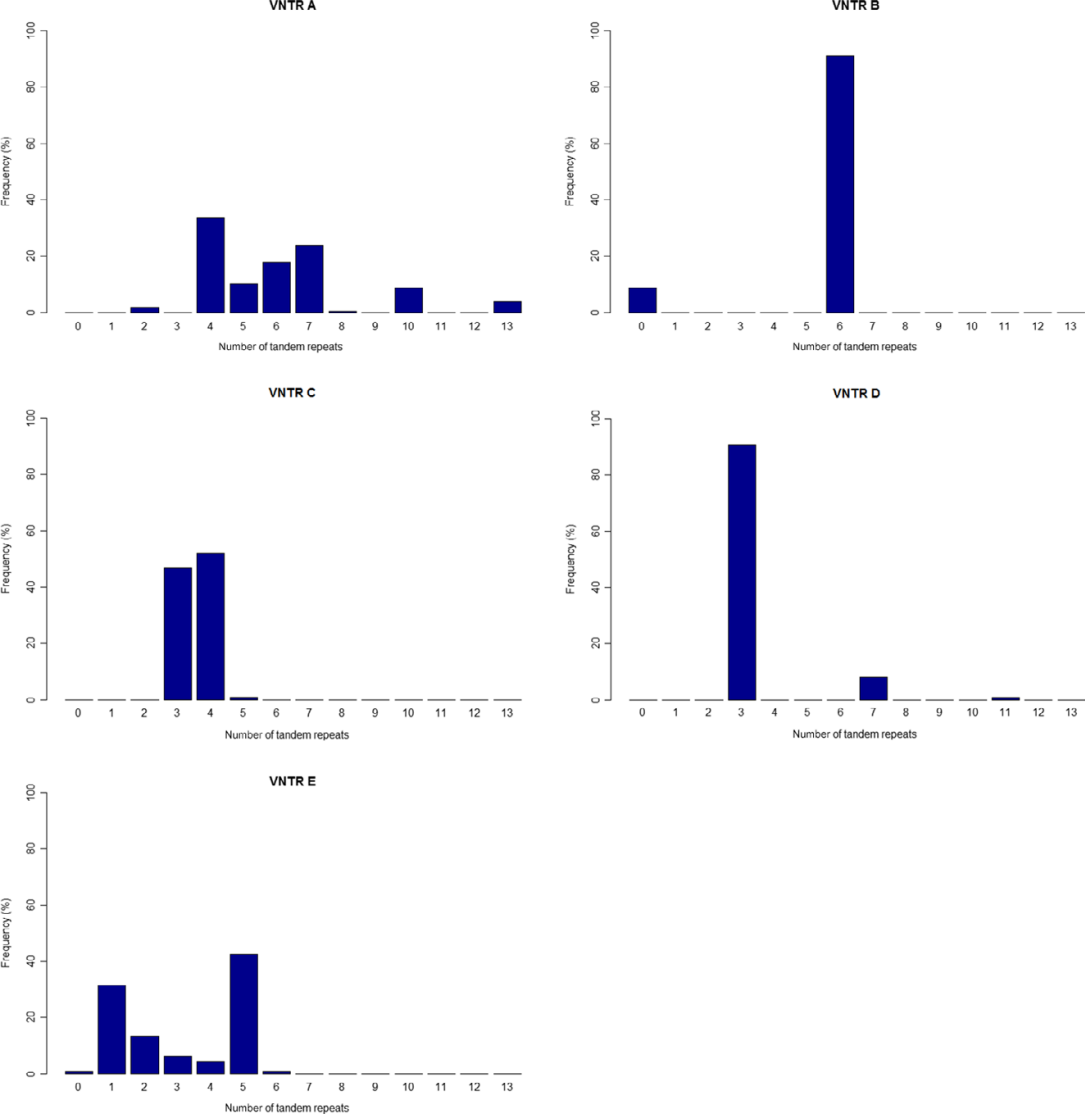
Diversity of VNTR loci. For each VNTR locus the tandem repeat (TR) copy numbers are given with their respective prevalence in our dataset. VNTR A: 8 alleles (2, 4, 5, 6, 7, 8, 10, 13 TR), VNTR B: 1 allele (6 TR) and no amplicon in 8.2% of the strains (the ERIC II and IV strains), VNTR C: 3 alleles (3, 4, 5 TR), VNTR D: 3 alleles (3, 7, 11 TR), VNTR E: 6 alleles (1, 2, 3, 4, 5, 6) and no amplicon in 0.5% of the strains (ERIC III strains).

VNTR A was found to be the most diverse locus, splitting up in 8 different lengths of tandem repeats. The number of tandem repeats ranged from 2 to 13. The second most divers VNTR was VNTR E, followed by VNTR C, D and B (Table 3 and Fig. 2). The discriminatory power of the MLVA is due to the complementary discriminating power of each VNTR locus. The MLVA typing method based on these VNTR loci is powerful enough to find new MLVA types for strains that are not present in the tested dataset.

**Table 3:** VNTR characteristics of the full dataset (193 isolates)

VNTR	Length TR	No. alleles	No. repeat copies	Min rep	Max rep	Shannon	Simpson
VNTR A	19	8	2, 4, 5, 6, 7, 8, 10, 13	2	13	1.68	0.78
VNTR B	21	1	6	6	6	0.29	0.16
VNTR C	24	3	3, 4, 5	3	5	0.74	0.51
VNTR D	24	3	3, 7, 11	3	11	0.34	0.17
VNTR E	68	6	1, 2, 3, 4, 5, 6	1	6	1.39	0.70

The length of the tandem repeat, the number of different alleles (No. alleles), the number of tandem repeats in each allele (No. repeat copies), the maximum and the minimum number of repeats (max rep, min rep) are given. The Shannon Index and Simpson Index were both used to measure the diversity.

### Evolutionary history

**Figure 3:**
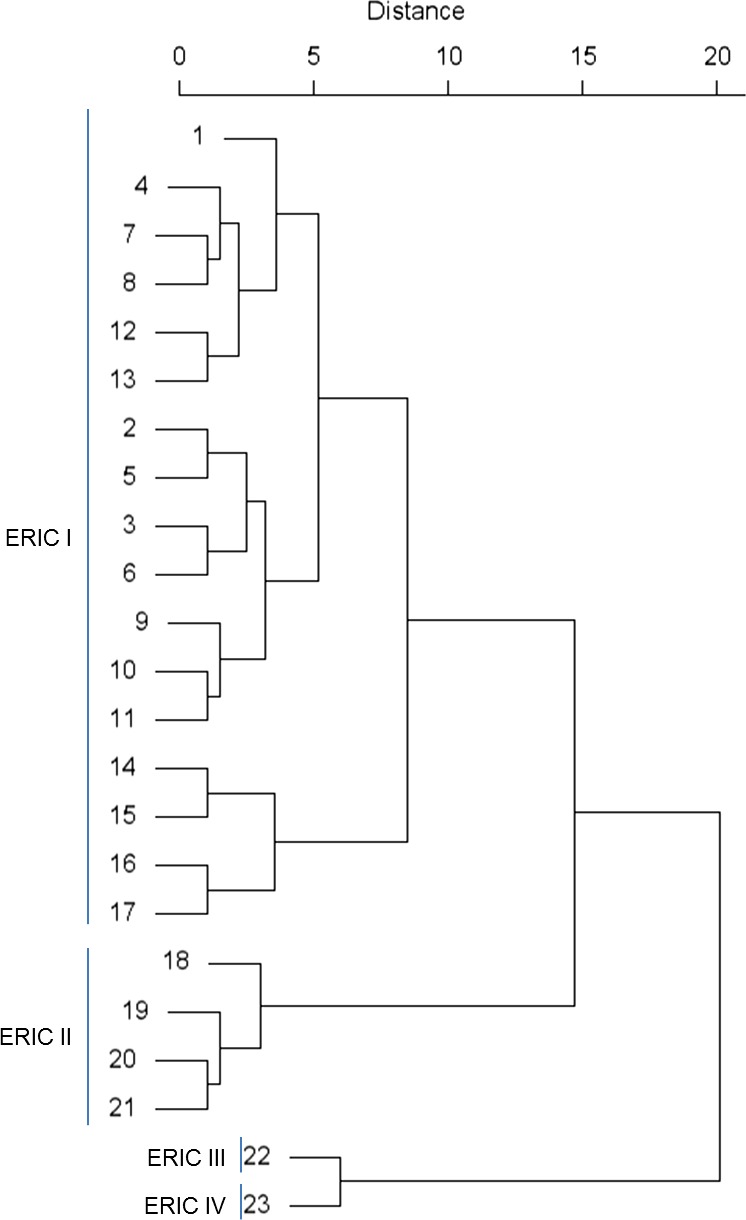
Evolutionary history of MLVA types. The evolutionary history of the MLVA types is based on hierarchical clustering. The distances were calculated using the sum of absolute differences in VNTR repeat number. The grouping reflected the ERIC‐typing method.

The five loci had all different molecular clock speeds, which makes it possible to look at the evolutionary history of the MLVA types (Fig. 3). The first node in the tree clustered the types 22 and 23 apart. To these MLVA types belong the ERIC type III and type IV strains respectively. These are the only strains of the tested collection that correspond to the former *P. larvae pulvifaciens* subspecies. The second node clustered all MLVA types that contained ERIC type I strains apart from the types that contained the ERIC type II strains.

### ERIC genotyping

All strains were also typed using ERIC rep‐PCR, which confirmed the presence of 189 ERIC type I strains, 18 ERIC type II strains, 1 ERIC type III and 1 ERIC type IV strain. Within ERIC type I, 17 MLVA genotypes could be discriminated and ERIC type II 4 MLVA genotypes were identified. Within ERIC type III and ERIC type IV, each time a single MLVA type could be attributed (Table 2). The subdivision of ERIC type I and ERIC type II in multiple subtypes is in the line of the findings of Morrissey (Morrissey *et al*., 2015) who found by MLST 16 subtypes in 173 ERIC type I strains and three subtypes in 92 ERIC II strains. Both typing schemes have thus on average the same resolution.

The present MLVA genotyping protocol also permits to differentiate between the ERIC genotypes by the presence of some discriminatory bands. Indeed, a band of 120 base pairs is always present in ERIC type I, but not in the ERIC type II. ERIC type III and type IV have a similar pattern, however, ERIC type III has always a band of 200 base pairs, which is absent in ERIC type IV (Fig. 1). The VNTR B primers do not create an amplicon in ERIC II and IV strains, while the VNTR E primers do not create an amplicon in ERIC II strains.

### Prevalence and biogeography

The prevalence of the MLVA types within the complete collection differed significantly (Table 2). Only three types had a prevalence of more than 10.0%: MLVA 2 (30.1%), MLVA 13 (17.7%) and MLVA 10 (11.0%). Within the ERIC II strains, MLVA 20 was predominant (14/18 isolates), this type contained isolates from Belgium, Italy and Austria. Almost half of the MLVA types (10 of 23) were represented by 1 or 2 isolates, corresponding to a prevalence of 0.5–1%.

**Figure 4:**
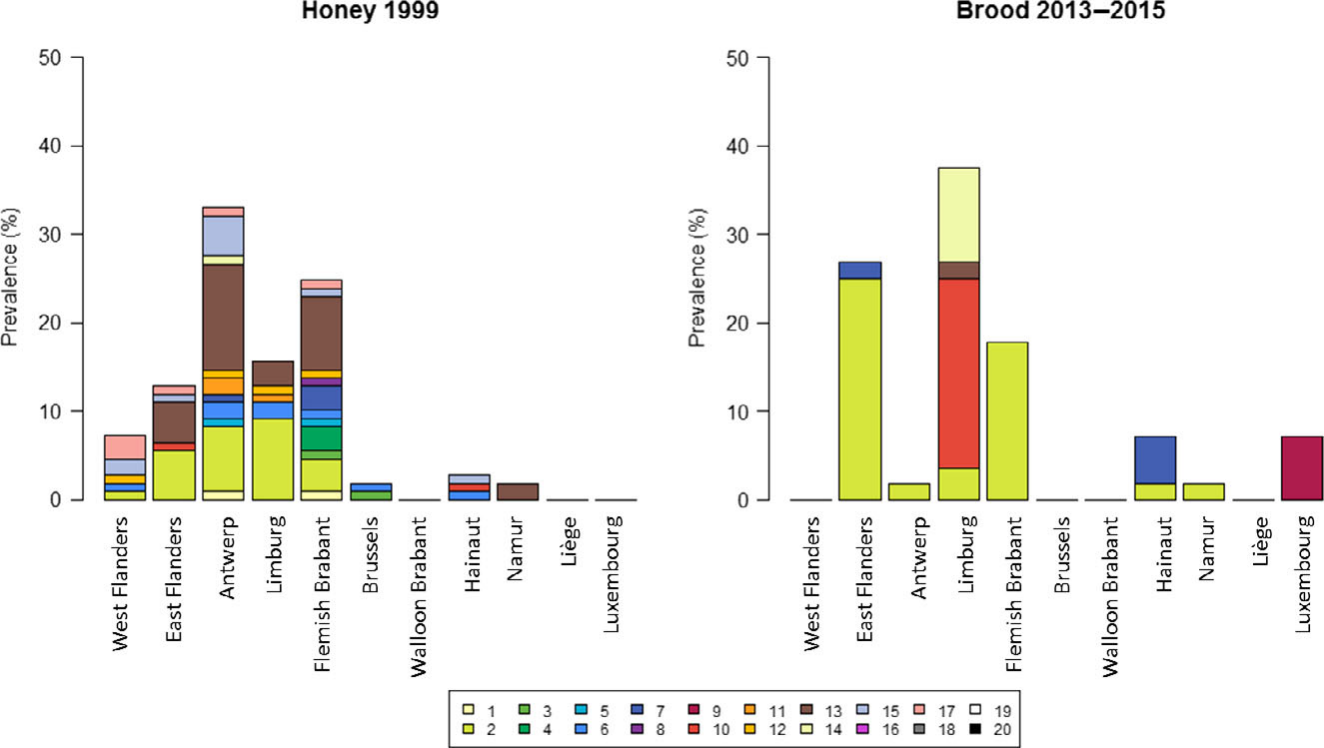
Distribution of MLVA‐types. The geographical location of each isolate from honey in 1999 and diseased brood in 2013–2015 was recorded. A significant difference in distribution over the provinces was observed. A much higher diversity in MLVA‐types was found in the honey samples than in the brood samples.

As the geographical spread of the apiaries where the isolates originated from (honey or brood samples) were mostly known (169 strains), we were able to provide the distribution per province (Fig. 4). A chi‐squared test using the geographical region (province) of the apiaries and the MLVA type was conducted and gave a very significant *P*‐value of < 0.001 (χ^2^ = 153.33, df = 144). We saw a much greater diversity in the subset of strains that originated from the honey screen compared with those from the clinical outbreaks (Shannon diversity index of 2.11 and 1.36, respectively). In fact, in the latter mostly 1 or 2 MLVA types were found per province, this could be due to the rapid spread of the spores in the vicinity of a clinical outbreak as described in de Graaf *et al*. (2001).

Remarkably, the MLVA type 2 which was most abundant in clinical cases in East Flanders, Antwerp, Limburg and Flemish Brabant and was likewise abundant in honey samples taken almost two decades earlier. It is not unravelled yet whether this is due to failure to eliminate the contamination with this MLVA type completely or to its biological characteristics (virulence). Another MLVA type (MLVA 13) that was abundant in honey samples of the same provinces, was almost entirely absent in the clinical cases of the period 2015–2013. In the brood samples of the province Limburg, MLVA 10 represents more than half of the isolates, while it is not present in any of the clinical cases of the other provinces.

**Figure 5:**
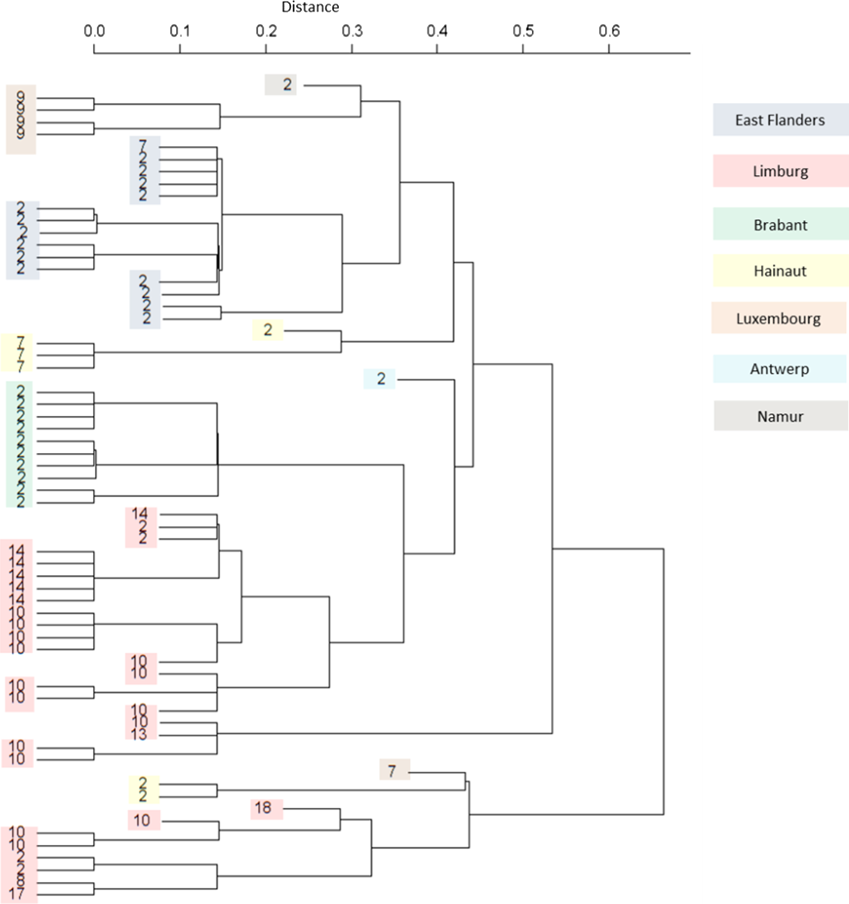
Agnes hierarchical clustering. Agnes hierarchical clustering based on the dissimilarities of non‐genetic traits clustered the strains by MLVA type and province. The correlation between these two parameters was visual.

Finally, it was investigated if classification of the isolates using a dissimilarity matrix resulted in the same MLVA types classified in the same group. Agglomerative hierarchical clustering was used as the algorithm. From the resulting tree (Fig. 5), we can conclude that indeed strains with the same MLVA type are on average more similar to each other based on non‐genetic traits (regional code, province, beekeeper, isolation year, isolation source) than to other MLVA types. The significant correlation with the region is also visual.

## Conclusions

In this paper, the development of a new genotyping method for *P. larvae* is presented. Using this method, we could subdivide ERIC type I into 17 different genotypes and ERIC type II into four different genotypes. ERIC type III and IV were represented each time by only a single MLVA genotype. Moreover, in the present MLVA genotyping protocol, amplicons indicative for the ERIC genotype were included, making it possible to extract information of the ERIC genotype from the MLVA pattern. This study clearly shows the usefulness of the MLVA method in epidemiological and biogeographical studies. MLVA genotyping has the unique combination of desirable properties for a genotyping method. It is very fast, highly specific, cheap and not labour intensive, which makes it an almost perfect method to implement in bee health extension laboratories. The method is usable for epidemiological, phylogenetic and evolutionary studies.

## Experimental procedures

### Dataset

In the library, 116 isolates were collected in the context of the honey screening in 1999 (de Graaf *et al*., 2001). This study was conducted anonymously throughout Belgium, with the postal code of the area where the apiary was located as geographical reference. One honey sample was collected per apiary. The library also consisted of isolates collected from diagnostic AFB cases in Belgium, including a set of isolates from clinical outbreaks from 2013 to 2015. From the latter, the postal code and beekeeper was recorded. Finally, the set of field isolates was completed with six Austrian ERIC type II isolates (Loncaric *et al*., 2009), 10 Italian ERIC type II isolates (Bassi *et al*., 2015), ERIC type III (LMG 16525), and ERIC type IV (LMG 16247) (both from the BCCM/LMG bacteria collection) reference strains, and a sequenced ERIC type I strain (BRL 230010) (Qin *et al*., 2006; Chan *et al*., 2011).

### Preparation dataset

Bacterial genomic DNA was prepared using InstaGene matrix (Bio‐Rad, Hercules, CA, USA) according to the protocol described by Genersch and Otten (Genersch and Otten, 2003). To confirm that the isolates belong to *P. larvae* a 16S rDNA PCR was performed as described by Dobbelaere (Dobbelaere *et al*., 2001). Of each strain, the ERIC genotype has been determined according to the protocol of Genersch and Otten (2003).

### VNTR prediction

The publically available genomes of BRL 230010 (Qin *et al*., 2006; updated by Chan *et al*., 2011), DSM25430 (Djukic *et al*., 2014) and DSM 25719 (Djukic *et al*., 2014) were used as input in the *Tandem Repeats database* (Gelfand *et al*., 2006). As selection criteria, we set the alignment parameters to match = 2, mismatch = 5, indels = 7, the minimal alignment score = 80, the pattern size ≤ 120 bp. From the obtained list of possible targets (Table S1), primers (IDT, Leuven, Belgium) were designed for loci that (i) had a different copy number in at least two genomes or (ii) had a pattern size between 15 and 30 or (iii) had a copy number of more than five units. The primers were constructed targeting the flanking regions of the tandem repeat locus and to have an annealing temperature of 52°C. The differentiating power of the tandem repeat loci was tested using 13 *P. larvae* isolates. Ten of the 13 isolates were randomly selected from the dataset, and were extended with R‐20833 (ERIC type II), LMG 16252 (ERIC type III) and LMG 16247 (ERIC type IV). A locus suitable to include in the multiplex PCR should generate a specific amplicon for each isolate and had to make discriminate between strains possible using agarose gel electrophoresis.

### Construction multiplex PCR

Seven loci were initially combined in a multiplex PCR. Two loci were omitted, because the first one generated off target amplicons and the second failed to generate amplicons. The multiplex PCR was optimized by testing a DNA‐concentration gradient (20–120 ng DNA), MgCl_2_ gradient (1–3 nM) and variable combinations of primer concentrations (0.2–4 μM).

The final multiplex PCR used the HotStarTaq *Plus* DNA Polymerase kit (Qiagen, Hilden, Germany) and consisted of 1× PCR buffer, 2.5 mM MgCl_2_, 1× Q‐solution, 400 μM dNTPs, 0.8 μM of each VNTR A primer (IDT), 0.4 μM of each VNTR B – D primer and 4 μM of each VNTR E primer (Table 1). The total reaction volume was 25 μl and 100 ng total DNA is used as template. The PCR‐programme was as followed: 5′ 95°C, 30× (1′ 94°C, 1′ 52°C, 1′ 72°C) and 10′ 72°C. The multiplex PCR was repeated three times on the 13 isolates and proved to give reproducible results.

### Data scoring and description profiles

The length and number of tandem repeats was determined by analysing the agarose gel and confirmed by sequencing the differing amplicons of the 23 MLVA types. Sequencing was done by Sanger sequencing performed by GATC‐Biotech (Constance, Germany). The number of repeat units was rounded to the next highest integer number. If more than one amplicon for a specific VNTR was detected and the difference in length between these amplicons was higher than the repeats length (stutter peaks), the amplicon with the most intense band was used to assign the repeat number. The Shannon and Simpson diversity index was calculated using R (R Core Team, 2015). Using agglomerative hierarchical clustering (Agnes), evolutionary analysis was performed. The clustering method used the sum of the absolute distances to calculate the dissimilarities between observations.

### Analysis correlations

All statistical analysis is performed using R (R Core Team, 2015). Correlations were analysed using a Pearson's Chi‐square test with the geographical location (province) and MLVA type as variables. To perform a reliable test, MLVA types with less than five isolates were removed, provinces containing less than five isolates were joined with neighbouring provinces. A Pearson's chi‐squared test was performed on the full dataset.

### Agnes classification

In a final stage of the epidemiological analysis, an Agnes classification was performed. This is a hierarchical classification and can be used to show how the different MLVA classes cluster together when the MLVA type is not taken into consideration. Since our variables are almost exclusively categorical variables, a dissimilarity matrix had to be constructed first. This numerical dissimilarity matrix was then be used as input in Agnes.

## Supporting information


**Table S1.** Tandem repeats of published genomes.
