# Supplementary material for: Multiple Locus Variable number of tandem repeat Analysis: A molecular genotyping tool for Paenibacillus larvae
Source: Microb Biotechnol. 2016 Jul 1;9(6):772–81. doi: 10.1111/1751-7915.12375 (PMC5072193; doi:10.1111/1751-7915.12375)
Supplement: Supplementary file 1 — Table S1. Tandem repeats of published genomes. [file MBT2-9-772-s001.docx]

**Supplementary material MLVA *paenibacillus***

Legend

Table S1 Tandem repeats of published genomes. With the *Tandem* *repeats* *database* tool (Gelfand et al., 2006), 40 different tandem repeat loci could be found in the 4 published genomes of *Paenibacillus larvae.* Of each locus the pattern size as found by the tool is given. For each genome the indices where the tandem repeat locus can be found is given together with the respective copy number. The genome sequences used as input are BRL230010 (Qin et al., 2009) and its update B3650 (Chan et al., 2011), DSM25430 and DSM25719 (Djukic et al., 2014).

Table S1

| Locus | pattern size | B3650 | | DSM25430 | | BRL230010 | | DSM25719 | |
| --- | --- | --- | --- | --- | --- | --- | --- | --- | --- |
|  |  | indices | copy nr | indices | copy nr | indices | copy nr | indices | copy nr |
| 1 (VNTR F) | 96 | 14006-14228 | 2,32 | 3748698-3749016 | 3,32 | 31114305-3114552 | 2,58 |  |  |
|  | 48 | 14059-14207 | 3,09 | 3748751-3748995 | 5,09 |  |  |  |  |
|  | 48 |  |  | 3748830-3748978 | 3,14 |  |  |  |  |
| 2 | 42 | 266373-266523 | 3,59 | 213152-213302 | 3,59 | 273771-273921 | 3,59 | 2416266-2416416 | 3,59 |
|  | 43 |  |  | 213059-213182 | 2,93 |  |  |  |  |
| 3 | 42 | 341012-341107 | 2,28 | 2329305-2329400 | 2,28 |  |  |  |  |
| 4 | 27 | 461373-461470 | 3,62 |  |  | 3871250-3871347 | 3,62 | 3295951-3296048 | 3,62 |
| 5 | 36 | 535762-535841 | 2,22 | 827383-827464 | 2,27 |  |  | 1822792-1822871 | 2,22 |
| 6 | 6 | 599877-599981 | 17,5 |  |  | 3966747-3933851 | 17,5 | 3990523-3990627 | 17,5 |
| 7 | 40 | 901066-901150 | 2,12 |  |  | 1887921-1888005 | 2,12 | 3848292-3848376 | 2,12 |
| 8 (VNTR A) | 19 | 932188-932289 | 5,36 | 399514-399615 | 5,36 | 424455-424613 | 8,36 | 2867714-2867815 | 5,36 |
| 9 | 36 | 954413-954526 | 3,16 | 2630403-2630516 | 3,16 | 3901081-3901203 | 3,43 | 1342002-1342115 | 3,16 |
| 10 | 28 | 1174279-1174339 | 2,18 |  |  | 447740-447803 | 2,28 | 1624897-1624960 | 2,28 |
| 11 | 84 | 1258490-1258706 | 2,65 | 2327560-237776 | 2,65 | 2652233-2652428 | 2,36 | 4528637-4528853 | 2,65 |
|  | 42 | 1258511-1258706 | 4,73 | 2327581-2327776 | 4,73 | 2652234-2652449 | 5,28 | 4528658-4528853 | 4,73 |
|  | 83 |  |  |  |  | 2652234-2652450 | 2,65 |  |  |
|  | 42 |  |  |  |  | 2652006-2652101 | 2,28 |  |  |
| 12 | 36 | 1500754-1500824 | 1,97 |  |  | 2292269-2292339 | 1,97 | 702952-703022 | 1,97 |
| 13 | 30 | 1549326-1549385 | 2 | 2680828-2680887 | 2 | 1006296-1006355 | 2 | 3316794-3316853 | 2 |
| 14 | 66 | 1560818-1561054 | 3,47 |  |  |  |  | 590200-590436 | 3,47 |
| 15 | 48 | 1699220-1699312 | 1,93 | 2799503-2799688 | 3,87 | 3155825-3156013 | 3,93 | 3436237-3436425 | 3,93 |
| 16 | 51 | 1743570-1743675 | 2,07 | 3418713-3418826 | 2,23 | 1667616-1667721 | 2,07 | 1749166-1749279 | 2,23 |
| 17 (VNTR G) | 18 | 1791546-1792111 | 32,94 | 690030-690627 | 35,22 | 3672843-3673699 | 50,1 | 4112219-4112694 | 27 |
|  |  |  |  |  |  | 16331-16588 | 14,8 |  |  |
|  | 36 | 1791546-1792118 | 16,67 |  |  | 3673013-3673712 | 2,16 |  |  |
|  | 99 | 1791555-1792118 | 5,88 |  |  |  |  | 4112228-4112694 | 4,65 |
|  | 9 |  |  | 689987-690622 | 70,61 |  |  |  |  |
|  | 89 |  |  | 689994-690622 | 7,17 |  |  |  |  |
|  | 81 |  |  | 690005-690624 | 7,59 |  |  |  |  |
|  | 80 |  |  | 690120-690751 | 7,96 |  |  |  |  |
|  | 45 |  |  | 690238-690726 | 11,56 | 16373-16559 | 4,14 |  |  |
|  | 27 |  |  |  |  | 16356-16577 | 8,23 |  |  |
| 18 | 78 | 2047981-2048193 | 2,73 | 2798732-2799023 | 3,74 | 3155057-3155348 | 3,74 | 3436902-3437192 | 2,48 |
|  | 39 | 2048006-2048191 | 4,76 | 2798732-2799022 | 7,46 | 3155057-3155347 | 7,46 | 3436903-3437193 | 7,46 |
|  | 117 |  |  | 2798732-2799023 | 2,49 | 3155057-3155348 | 2,49 | 3436902-3437192 | 2,48 |
| 19 | 47 | 2113412-2113519 | 2,29 |  |  |  |  |  |  |
| 21 | 36 | 2348185-2348255 | 1,97 |  |  |  |  |  |  |
| 22 | 48 | 2348975-2349138 | 3,41 |  |  | 2533998-2534161 | 3,41 | 4190293-4190456 | 3,41 |
|  | 27 | 2348990-2349141 | 6,07 |  |  | 2534094-2534146 | 1,96 | 4190308-4190459 | 6,07 |
| 23 | 63 | 2357340-2357475 | 2,15 |  |  | 456088-456223 | 2,15 | 4354854-4354989 | 2,15 |
| 24 (VNTR C) | 48 | 2595946-2596108 | 3,39 | 3429304-3429458 | 3,17 | 1704713-1704842 | 2,7 | 425682-425811 | 2,7 |
|  | 24 | 2596009-2596088 | 5,33 |  |  | 1704715-1704842 | 5,33 | 425684-425811 | 5,33 |
| 25 | 15 | 2886203-2886252 | 3,33 | 833249-833328 | 5,25 | 3050585-3050634 | 3,33 | 1097173-1097222 | 3,33 |
|  | 31 |  |  | 833249-833318 | 2,29 |  |  |  |  |
| 26 | 45 | 3005137-3005355 | 4,84 |  |  |  |  | 3133078-3133296 | 4,84 |
| 27 | 42 | 3403722-3403806 | 2,07 |  |  |  |  | 319288-3192968 | 2,07 |
| 28 | 18 |  |  | 109356-109415 | 3,33 |  |  |  |  |
| 29 (VNTR E) | 68 |  |  | 1647396-1647833 | 6,46 |  |  |  |  |
| 30 | 120 |  |  | 2907751-2908124 | 3,12 | 681789-682163 | 3,12 | 1187984-1188358 | 3,12 |
| 31 | 29 |  |  | 3332560-3332625 | 2,27 |  |  |  |  |
| 32 | 96 |  |  | 3393455-3393634 | 1,87 |  |  | 2163806-2164111 | 3,18 |
|  | 69 |  |  | 3393572-3393705 | 1,94 |  |  | 1724662-1724790 | 1,86 |
|  | 48 |  |  |  |  |  |  | 2163846-2164090 | 5,09 |
| 33 | 45 |  |  | 3475347-3475790 | 9,84 |  |  |  |  |
| 34 | 45 |  |  | 3487920-3488015 | 2,13 |  |  |  |  |
| 35 (VNTR B) | 42 |  |  |  |  | 2683910-2683997 | 2,09 |  |  |
|  | 21 |  |  |  |  | 2683932-2683997 | 3,14 |  |  |
| 36 | 36 |  |  |  |  | 3065163-3065244 | 2,27 |  |  |
| 37 | 31 |  |  |  |  | 3407625-3407686 | 2,03 |  |  |
| 38 | 69 |  |  |  |  | 3415180-3415308 | 1,86 |  |  |
| 39 | 18 |  |  |  |  | 3137833-3138128 | 16,44 | 457398-4574227 | 13,44 |
|  | 36 |  |  |  |  |  |  |  | 8,16 |
| 40 (VNTR D) | 24 |  |  | 3429304-3429458 | 6,33 |  |  |  |  |
